# Physicians' Earnings Do Not Affect Their Online Ratings

**DOI:** 10.3389/fpubh.2020.00300

**Published:** 2020-07-09

**Authors:** Sean C. Haffey, Wilma M. Hopman, Michael J. Leveridge

**Affiliations:** ^1^Department of Family Medicine, Queen's University, Kingston, ON, Canada; ^2^Department of Public Health Sciences, Kingston Health Sciences Centre, Queen's University, Kingston, ON, Canada; ^3^Department of Urology, Kingston Health Sciences Centre, Queen's University, Kingston, ON, Canada

**Keywords:** patient satisfaction, physician income, online rating sites, doctor-patient relationships, internet

## Abstract

**Objective:** Physician-rating websites have exploded in popularity in recent years. Consequently, these sites have garnered attention from researchers interested in factors influencing patient satisfaction. A doctor's earnings might reflect practice patterns that could influence their patients' perceptions. We sought to explore any association between physicians' earnings and their online ratings.

**Methods:** The names and billings of 500 physicians from British Columbia, Canada were randomly extracted from the 2016-17 *BC Blue Book* and matched to their profiles on *RateMDs.com*. Physicians' earnings were compared to their global ratings and to their Staff, Punctuality, Helpfulness, and Knowledge scores. Earnings and ratings were also compared between men and women, as well as between family medicine, surgical, and internal medicine and subspecialties cohorts.

**Results:** We found no significant correlation between physicians' earnings and their global online ratings (*p* = 0.304). Weak negative correlations existed between earnings and Staff and Helpfulness ratings (Spearman's rho = −0.055, *p* < 0.001; rho = −0.033, *p* < 0.028). Online ratings were largely favorable (mean MD rating of 3.85/5. Male physicians earned significantly more than their female colleagues ($371,734.85 and $261,590.82, respectively; *p* < 0.001), but no significant difference existed between men and women with regards to online ratings (mean 3.87 and 3.81, respectively, *p* = 0.191). Surgical and Family Medicine specialties showed a negative correlation between income and ratings; no relationship was seen in the internal medicine and subspecialties cohort.

**Conclusions:** No meaningful association was found between physicians' earnings and their online ratings, although there is an impact of specialty grouping. Patients tend to review doctors favorably online; these data add to the discussion of whether male and female doctors are differentially rated. Trends toward increased transparency in health care systems may help to elucidate how doctors' earnings influence patients' perception of and satisfaction with the care they receive.

## Introduction

Websites dedicated to rating and evaluating physicians have become increasingly popular over the past decade. By providing a virtually anonymous platform for patients to rate and comment on medical professionals, these public reporting forums are now used by patients in a similar manner and frequency as online consumers of movies, cars and restaurants (1). While the majority of patients who use physician-rating websites tend to be younger, more digitally literate individuals with higher levels of education, their decisions on whether or not to seek care from a particular physician are becoming increasingly influenced by decidedly positive (or negative) online reviews (2–5).

The physician-rating website *RateMDs.com* is particularly popular in Canada and the United States. This free online rating tool, which exclusively evaluates health care providers, contains over 2.6 million physician reviews and has served over 161 million users since its founding in 2004 (6, 7). While published estimates of what proportion of doctors were rated on the website at the beginning of the decade range from 5 to 27%, medical researchers are nevertheless beginning to recognize the popularity and, by extension, potential utility of this physician rating website (8–11). For instance, contrary to the previous reports that physician-rating websites are largely predominated by poor reviews from disgruntled patients, more recent studies show that doctors' online ratings in fact tend to be favorable and correlate with parameters measuring physician quality, such as experience, level of education, and fewer malpractice claims (9, 12–14). Consequently, websites like *RateMDs.com* are gaining recognition as useful sources of information for researchers interested in quality improvement in health care, especially through the lens of patient satisfaction (4).

Despite a growing research interest in factors that impact physician ratings online, one factor that remains unexplored is physician income. A doctor's earnings could influence how their patients perceive them online for a number of reasons. Firstly, income could serve as a surrogate for time spent with patients in a fee-for-service environment. This would presumably be lower in doctors who hold busier clinics and resultantly bill more. Conversely, high billing could be a reflection of esteem, merit, skill, specialty, or of long working hours spent caring for patients, all of which may be highly rated. We hypothesized that physicians' annual billing income would impact both their overall online ratings, as well as sub-ratings that address physicians' staff, punctuality, helpfulness, and medical knowledge.

## Methods

The names and 2016-17 gross billings of all 3,720 health care professionals listed in the *BC Blue Book* were extracted using Tabula software (15). Individuals were then assigned identification numbers from 0001 to 3720 based on their surname. An online random number generator, GraphPad QuickCalcs, produced a list of random numbers from 1 to 3720, and the corresponding physicians and their incomes were selected in order. We sought a sample of 500 physicians, which required the generation of 859 random numbers. We excluded physicians billing under $50,000 in the 2016-17 year (103), as well as those who were not medical doctors (27; e.g., physiotherapists, dentists, midwives, etc.) or for whom a RateMDs profile could not be found (229). In total, 500 physicians randomly selected from the *BC Blue Book* and matched to a RateMDs profile were included in our analysis.

Based on their online profile, each physician's gender, number of reviews and global (i.e., mean) rating were recorded. In addition, each physician's most recent individual reviews (up to a maximum of 10) were recorded along with their four constituent scores: Staff, Punctuality, Helpfulness and Knowledge. Both the global and constituent scores were ranked on an ordinal one-to-five ‘stars' scale. Of note, no numerical global ranking is provided on RateMDs, and so these were ascertained from the graphical representation of physicians' “star” ratings.

Data were imported into IBM SPSS (version 24.0 for Windows, Armonk, New York, 2016) for statistical analysis. The data were initially analyzed descriptively, including frequencies and percentages for categorical data and means and standard deviations for the ordinal and continuous data. Independent samples t-tests were used to compare men and women, and Spearman correlation was used to examine the association between income and the rating of staff, punctuality, helpfulness, and knowledge. Specialty was grouped for analysis into family medicine, internal medicine and subspecialties and surgical specialties and compared using one-way analysis of variance (ANOVA). A *p*-value of 0.05 was used as the criterion for statistical significance.

## Results

**Figure 1:**
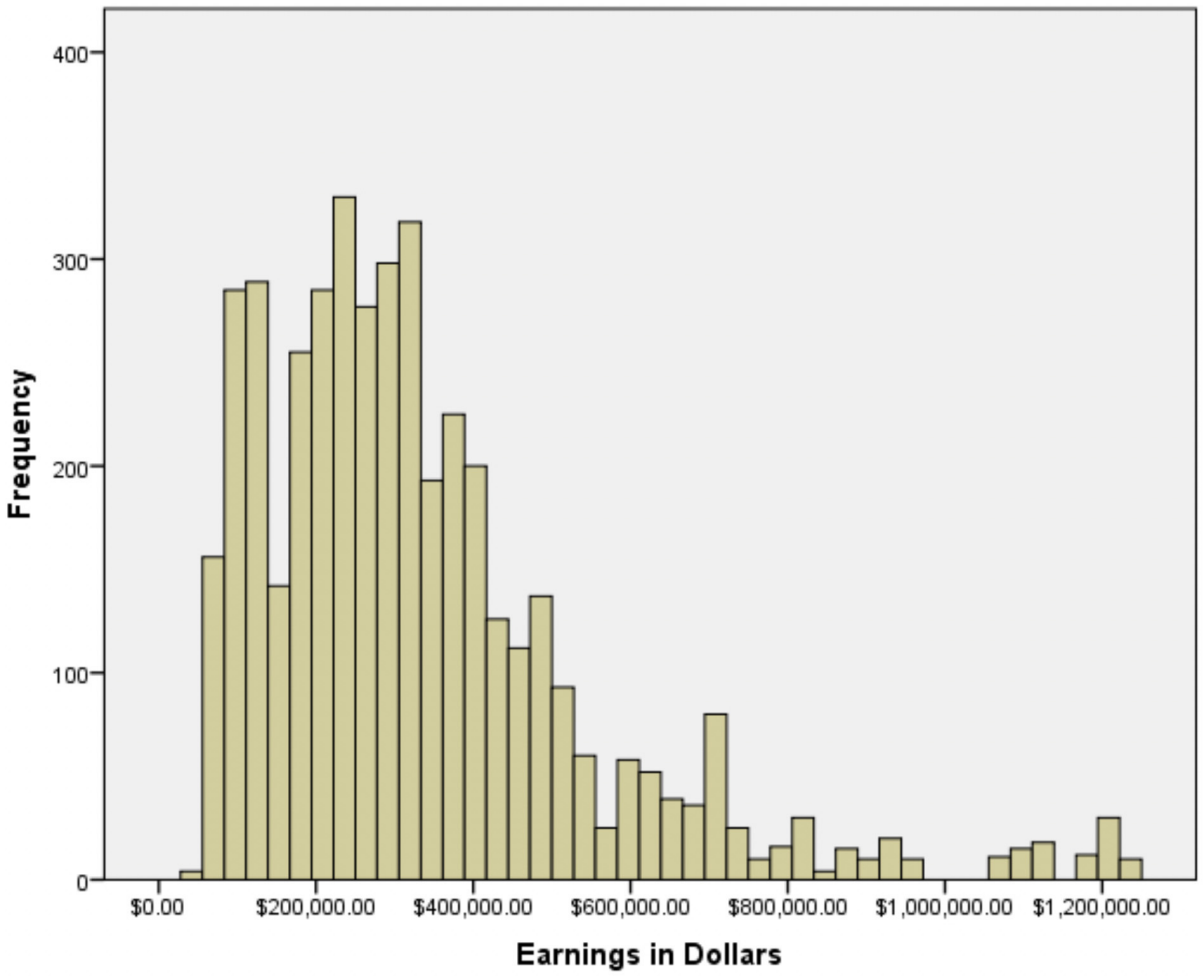
Distribution of billings.

Of the 500 physicians included in our analysis, 66.4% were male (332) and 33.7% were female (168), slightly skewed from the 2018 totals from British Columbia (61.8% male and 38.2% female) (16). Billings ranged from $51,645.26 to $3,006,923.08 (Figure 1). Of these, four were outliers (defined as three standard deviations beyond the mean physician income of $352,846.69) and were excluded from further analyses. Physicians garnered an average global rating of 3.85 and average Staff, Punctuality Helpfulness, and Knowledge ratings of 3.87, 3.76, 3.83, and 3.94, respectively (Table 1). Male physicians earned significantly more than their female colleagues ($371,734.85 and $261,590.82, respectively; *p* < 0.001, 95% CI for difference $118,228.64 – $153,809.25; Table 2). Despite the gap, however, no significant difference existed between men and women with regards to mean online ratings (3.87 and 3.81, respectively, *p* = 0.191). We found no significant correlation between physicians' earnings and their global online ratings (*p* = 0.304, Table 3). Weak negative correlations existed between Staff and Helpfulness ratings and physicians' earnings (Spearman's rho = −0.055, *p* < 0.001; rho = −0.033, *p* < 0.028). The ratings themselves (staff, punctuality, helpfulness, and knowledge) were all highly significantly correlated with each other, as one would expect, with rho values ranging from 0.697 (staff rating with knowledge rating) to 0.928 (helpfulness rating with knowledge rating), all *p* < 0.001.

**Table 1:** Descriptive statistics.

	***N***	**Minimum**	**Maximum**	**Mean**	**Std. Deviation**
**Descriptive statistics**					
Earnings in dollars	496	$51,645.26	$1,228,088.87	$335,147.01	$217,542.31
Staff rating	4238	1	5	3.87	1.48
Punctuality rating	4311	1	5	3.76	1.50
Helpfulness rating	4311	1	5	3.83	1.70
Knowledge rating	4311	1	5	3.94	1.60
Average of the 4 ratings	4311	1.00	5.00	3.85	1.43

**Table 2:** Differences in earnings and ratings between men and women.

	**Gender**	***N***	**Mean**	**Standard Deviation**	***P* (2-tailed)**
Earnings in dollars	Male	329	$371,734.85	$245,412.14	<0.001
	Female	167	$261,590.82	$152,140.00	
Staff rating	Male	2831	3.89	1.48	0.326
	Female	1398	3.84	1.49	
Punctuality rating	Male	2878	3.78	1.49	0.199
	Female	1424	3.72	1.50	
Helpfulness rating	Male	2878	3.84	1.69	0.337
	Female	1424	3.79	1.72	
Knowledge rating	Male	2878	3.96	1.59	0.111
	Female	1424	3.88	1.62	
Average of the 4 ratings	Male	2878	3.87	1.43	0.191
	Female	1424	3.81	1.43	

**Table 3:** Spearman correlations between earnings and ratings, overall and by specialty group.

		**Staff**	**Punctuality**	**Helpfulness**	**Knowledge**	**Average of the 4 ratings**
Earnings in dollars entire sample	Correlation coefficient	−0.055^**^	0.002	−0.033^*^	-0.021	−0.016
	*P* (2-tailed)	<0.001	0.907	0.028	0.170	0.304
	*N*	4238	4311	4311	4311	4311
Family medicine	Correlation coefficient	−0.096^**^	−0.053^**^	−0.101^**^	−0.092^**^	−0.079^**^
	*P* (2-tailed)	<0.001	0.009	<0.001	<0.001	<0.001
	*N*	2353	2412	2412	2412	2412
Internal medicine and specialties	Correlation coefficient	0.003	0.054	0.112^**^	0.080^*^	0.061
	*P* (2-tailed)	0.935	0.117	0.001	0.021	0.081
	*N*	828	832	832	832	832
Surgical specialties	Correlation coefficient	−0.138^**^	-0.093^*^	−0.048	-0.032	−0.113^**^
	*P* (2-tailed)	0.001	0.028	0.258	0.453	0.007
	*N*	563	563	563	563	563

** *Correlation is significant at the 0.01 level (2-tailed)*.

* *Correlation is significant at the 0.05 level (2-tailed)*.

Additional analyses were carried out between the 271 family medicine, 104 internal medicine and subspecialties and 61 surgical subspecialties. Not all specialties could be assigned a group (e.g., Emergency Medicine, Pediatrics, and Anesthesiology did not clearly fall into one group) so 60 of the 496 physicians were not included in this analysis. There were significant differences in income between these groups (Table 4). In each case, ratings were statistically significantly different, with mean ratings of 3.84, 3.69, and 4.05 respectively. In the family medicine cohort, ratings decreased with increasing income (Table 3). No relationship was found in the internal medicine cohort, and a weak negative association between income and rating was seen in the surgical cohort.

**Table 4:** Differences in earnings and ratings between specialties.

		***N***	**Mean**	**Standard Deviation**	***P* (2-tailed)**
Earnings in dollars	Family medicine	271	268,637.89	152,252.46	<0.001
	IM and specialties	104	404,509.14	247,442.80	
	Surgical specialties	61	479,837.37	305,515.35	
Staff rating	Family medicine	2353	3.86	1.46	0.001
	IM and specialties	828	3.75	1.58	
	Surgical specialties	563	4.06	1.42	
Punctuality rating	Family medicine	2412	3.70	1.49	<0.001
	IM and specialties	832	3.70	1.54	
	Surgical specialties	563	3.98	1.49	
Helpfulness rating	Family medicine	2412	3.86	1.67	<0.001
	IM and specialties	832	3.57	1.81	
	Surgical specialties	563	3.99	1.62	
Knowledge rating	Family medicine	2412	3.93	1.59	<0.001
	IM and specialties	832	3.74	1.71	
	Surgical specialties	563	4.18	1.44	
Average of the 4 ratings	Family medicine	2412	3.84	1.40	<0.001
	IM and Specialties	832	3.69	1.54	
	Surgical specialties	563	4.05	1.39	

## Discussion

Similar to business patrons seeking online ratings for products and services, patients increasingly turn to physician-rating websites to assess prospective doctors. Consequently, health care researchers are beginning to investigate these websites as potential vehicles for quality improvement. Several studies have analyzed the demographics of patients using physician rating websites, how highly (or poorly) they generally score physicians, and what exactly influences a doctor's ratings. These data replicate previous findings that online ratings of doctors tend to be favorable (8, 9, 11, 14).

Terlutter et al. assessed the demographics of patients using physician rating sites, and found a skew toward female gender, increasing education, and self-ascribed presence of chronic disease (2).

We calculated an average global rating of 3.85 stars on a 5-star scale, while Kadry and colleagues reported an average of 3.84 stars across ten separate rating websites that use similar scales (14). Additionally, we found no significant difference in ratings between male and female physicians, which both validates and contradicts previous findings. Some studies report gender equality in online ratings, while others assert that either male or female physicians boast superior ratings (9, 17–19). Regardless, these studies focused on different physician specialties and were based out of different countries, and so future studies may adapt a more international, pan-specialty approach in order to determine how physician gender truly affects online ratings.

We found little to no overall correlation between physician billing and online ratings. There may have been some offsetting of results based on disparities seen between specialty groupings. Although Staff and Helpfulness ratings did negatively correlate with income, these associations were very weak and likely uninformative (rho = −0.055, −0.033, respectively). These results are puzzling given that other physician qualities associated with higher earnings, such as prestige and particular specializations, have been shown to affect ratings. For instance, McGrath and colleagues recently reported that patients tend to give more favorable reviews to doctors listed on Castle Connolly Medical's “America's Top Doctors,” a peer-reviewed title that carries a sense of prestige or, at the very least, esteem (20). Moreover, surgical subspecialties often associated with higher incomes tend to receive higher ratings than their generalist counterparts (21, 22). In our cohort we did not replicate these findings, as there was a small negative correlation between income and ratings in the surgical cohort. Certain treatment modalities and diagnoses may also influence ratings, as patients receiving treatment for cancer or those actually receiving surgery tend to give surgical specialists higher ratings (23). The interplay between specialty, prestige and income is complex; further work is required to delineate their effects on patient satisfaction.

Ours is the first study to investigate the relationship between online ratings and physician billings. Although our findings do not suggest a clear relationship between the two, physician income is becoming a highly contentious issue in Canada, particularly with regards to the publishing of doctors' earnings. British Columbia, the focus of this study, is one of only two provinces that annually publish the names of all physicians and the amounts they bill their provincial insurance plans. This shadows a national trend aimed at increased transparency in the health care system using information published by the Canadian Institute for Health Information (24). Based on these data, some reports have contended that different physician payment models, either concurrent in the same health care system, or separated either geographically by country or temporally by future possible legislation, may uncouple income from the individual doctor-patient interaction and would undoubtedly alter expectations regarding physician ratings (25, 26). With other provinces being pressured to publish physicians' salaries (27), more information on the matter will likely become available in the coming years, which may shed more light on how physician income affects online ratings.

The present study carries limitations that merit consideration. Firstly, we only compared physicians' online ratings to their earnings reported in the 2016-17 year. This cross-sectional view of physicians' earnings does not capture changes over time that may reflect meaningful changes in practice. Indeed, a physician's online ratings may correlate differently with their earnings on a five- or ten-year scale than with those of a single year. Second, we did not take the narrative content in patients' reviews into consideration. This may have masked potential confounders, as physicians whose online profiles are dominated by fact-based reviews tend to receive higher ratings from new patients than those featuring more emotionally charged ones (4). It is impossible to verify the objectivity and veracity of individual reviews, though studies referenced above have used available data to show other factors outside the physician-patient relationship that may impact ratings. This contributes to the importance of studies such as this in the review space, to identify such factors. Finally, our study only included physicians from British Columbia, and therefore is not generalizable to the rest of Canada or other countries and health care systems. Therefore, future studies that incorporate broader practice type and health systems will be essential to delineating the factors that determine physicians' online ratings.

In conclusion, there is little association between physicians' income and their online ratings. Our study replicates previous findings such as the general favorability of patients' online reviews of their doctors, and adds to the data on gender differences in online ratings. We expect that emerging trends of increasing transparency will lead to more detailed information concerning physicians' practices; this will in turn help to elucidate how this information impacts patient perception.

## Data Availability Statement

The datasets generated for this study are available on request to the corresponding author.

## Author Contributions

SH and ML: data gathering and concept. WH, SH, and ML: data analysis, manuscript preparation, manuscript revision, accountability for work, and approval for publication.

## Conflict of Interest

The authors declare that the research was conducted in the absence of any commercial or financial relationships that could be construed as a potential conflict of interest.

## References

[1] Haunauer DA Zheng K Singer DC. Public awareness, perception, and use of online physician rating sites. JAMA. (2014) 311:734–5. 10.1001/jama.2013.283194 24549555

[2] Terlutter R Bidmon S Röttl J. Who uses physician-rating websites? Differences in sociodemographic variables, psychographic variables, and health status of users and nonusers of physician-rating websites. J Med Internet Res. (2014) 16:e97. 10.2196/jmir.3145 24686918 PMC4004145

[3] Burkle CM Keegan MT. Popularity of internet physician rating sites and their apparent influence on patients' choices of physicians. BMC Health Serv Res. (2015) 15:416. 10.1186/s12913-015-1099-2 26410383 PMC4583763

[4] Grabner-Kräuter S Waiguny MK. Insights into the impact of online physician reviews on patients' decision making: randomized experiment. J Med Internet Res. (2015) 17:e93. 10.2196/jmir.3991 25862516 PMC4408377

[5] Rothenfluh F Germeni E Schulz PJ. Consumer decision-making based on review websites: Are there differences between choosing a hotel and choosing a physician? J Med Internet Res. (2016) 18:e129. 10.2196/jmir.5580 27311623 PMC4929347

[6] RateMDs.com. The Original Doctor Rating and Review Site. (2020). Available online at: http://www.ratemds.com/about (Accessed May 27, 2020).

[7] Tanne J. How patients rate doctors. BMJ. (2008) 337:a1408. 10.1136/bmj.a1408 18826978

[8] Lagu T Hannon NS Rothberg MB Lindenauer PK. Patients' evaluations of health care providers in the era of social networking: An analysis of physician-rating websites. J Gen Intern Med. (2010) 15:942–6. 10.1007/s11606-010-1383-0 20464523 PMC2917672

[9] Gao GG McCullough JS Agarwal R Jha AK. A changing landscape of physician quality reporting: analysis of patients' online ratings of their physicians over a 5-year period. J Med Internet Res. (2012) 14:e38. 10.2196/jmir.2003 22366336 PMC3374528

[10] Mostaghimi A Crotty BH Landon BE. The availability and nature of physician information on the Internet. J Gen Intern Med. (2010) 25:1152–6. 10.1007/s11606-010-1425-7 20544300 PMC2947633

[11] Emmert M Sander U Pisch F. Eight questions about physician-rating websites: a systematic review. J Med Internet Res. (2013) 15:e24. 10.2196/jmir.2360 23372115 PMC3636311

[12] McCartney M. Will doctor rating sites improve quality of care? No. BMJ. (2009) 338:b1003. 10.1136/bmj.b1003 19293224

[13] Jain S. Googling ourselves–what physicians can learn from online rating sites. N Engl J Med. (2010) 362:6–7. 10.1056/NEJMp0903473 20054044

[14] Kadry B Chu LF Kadry B Gammas D Macario A. Analysis of 4999 online physician ratings indicates that most patients give physicians a favourable rating. J Med Internet Res. (2011) 13:e95. 10.2196/jmir.1960 22088924 PMC3222200

[15] DataBC Ministry of Health - Medical Services. BC Blue Book 2016-17. (2020). Available online at https://www2.gov.bc.ca/assets/gov/health/practitioner-pro/medical-services-plan/blue-book-2016-17.pdf (Accessed May 27, 2020).

[16] BC College of Physicians and Surgeons Annual Report 2016/7. (2020). Available online at: https://www.cpsbc.ca/annual-report/2017 (Accessed May 27, 2020)

[17] Trehan SK DeFrancesco CJ Nguyen JT Charalel RA Daluiski A. Online patient ratings of hand surgeons. J Hand Surg Am. (2016) 41:98–103. 10.1016/j.jhsa.2015.10.006 26710742

[18] Frost C Mesfin A. Online reviews of orthopedic surgeons: an emerging trend. Orthopedics. (2015) 38:e257–62. 10.3928/01477447-20150402-52 25901617

[19] Emmert M Meier F. An analysis of online evaluations on a physician rating website: evidence from a German public reporting instrument. J Med Internet Res. (2013) 15:e157. 10.2196/jmir.2655 23919987 PMC3742398

[20] McGrath RJ Priestley JL Zhou Y Culligan PJ. The validity of online patient ratings of physicians: Analysis of physician peer reviews and patient ratings. J Med Internet Res. (2018) 7:e8. 10.2196/ijmr.9350 29631992 PMC5913572

[21] Liu JJ Matelski JJ Bell CM. Scope, breadth and differences in online physician ratings related to geography, specialty, and year: Observational retrospective study. J Med Internet Res. (2018) 20:e76. 10.2196/jmir.7475 29514775 PMC5863010

[22] Daskivich T Luu M Noah B Fuller G Anger J Spiegel B. Differences in online consumer ratings of health care providers across medical, surgical, and allied health specialties: Observational study of 212,933. J Med Internet Res. (2018) 20:e176. 10.2196/jmir.9160 29743150 PMC5980486

[23] Ferrara S Hopman WM Leveridge M Diagnosis, bedside manner and comment style are predictive factors in online ratings of urologists. Urology Practice. (2014) 1:117–21. 10.1016/j.urpr.2014.05.005 37537827

[24] Collier R. Physician income: a look behind the numbers. CMAJ. (2015) 187:396. 10.1503/cmaj.109-4997 25712948 PMC4387028

[25] Collier R. Professionalism: how payment models affect physician behaviour. CMAJ. (2012) 184:E645–6. 10.1503/cmaj.109-4250 22847970 PMC3447070

[26] Hendrickson MA. Pay for performance and medical professionalism. Qual Manag Health Care. (2008) 17:9–18. 10.1097/01.QMH.0000308633.81979.7b 18204373

[27] Taber J Ontario Urged to Make Doctors' Compensation Public. Toronto, ON: The Globe and Mail (2018) Available online at: https://www.theglobeandmail.com/news/national/ontario-urged-to-make-doctors-pay-public/article29829785/ (Accessed May 27, 2020).

